# Extracellular vesicle proteomes reflect developmental phases of *Bacillus subtilis*

**DOI:** 10.1186/s12014-016-9107-z

**Published:** 2016-03-09

**Authors:** Yeji Kim, Nathan Edwards, Catherine Fenselau

**Affiliations:** Department of Chemistry and Biochemistry, University of Maryland, College Park, MD 20742 USA; Department of Biochemistry and Molecular and Cellular Biology, Georgetown University Medical Center, Washington, DC 20057 USA

**Keywords:** Label-free quantification, *Bacillus subtilis*, Secretome, Sporulation, Extracellular vesicles

## Abstract

**Background:**

Extracellular vesicles (EV) are spherical membrane-bound vesicles with nano-scale diameters, which are shed to the extracellular region by most eukaryotic and prokaryotic cells. Bacterial EV are proposed to contribute to intercellular communication, bacterial survival and human pathogenesis as a novel secretion system. EV have been characterized from many Gram-negative species and, more recently, from several vegetative Gram-positive bacteria. Further characterization of EV and their molecular cargos will contribute to understanding bacterial physiology and to developing therapeutic approaches.

**Results:**

*Bacillus subtilis* were observed to release EV to a similar extent during sporulation as during the vegetative growth phase. However, the two vesicular cargos show qualitatively and quantitatively different proteomes. Among 193 total proteins identified across both samples, 61 were shown to be significantly more abundant in EV shed by sporulating cells, with (log) ratio of spectral counts R_SC_ > 1 and Fisher-exact test FDR < 5 %. Sixty-two proteins were found to be significantly more abundant in EV shed by vegetative cells. Membrane fusion was shown to take place between these EVs and Gram-positive cells.

**Conclusion:**

Biogenesis of EV is a continuous process over the entire life cycle of this sporulating bacterium. The formation of EV during sporulation is strongly supported by the delineation of protein content that differs from the proteome of EV formed by vegetative spores.

**Electronic supplementary material:**

The online version of this article (doi:10.1186/s12014-016-9107-z) contains supplementary material, which is available to authorized users.

## Background

Extracellular vesicles (EV) are universally produced from diverse eukaryotes and prokaryotes [1, 2]. They are spherical and membranous vesicles shed to the extracellular region, and enclosed by a lipid bilayer with a nano-scale diameter between 20 and 1000 nm depending on the organism [3, 4]. As in the case with EV shed by multicellular organisms, Gram-negative bacterial EV carry diverse cell-derived components, including numerous proteins, lipids, genetic materials, toxins, communication signals, and immunomodulatory compounds [4–6]. EV have been proposed to contribute to intercellular communication, bacterial survival, and human pathogenesis as a novel secretion system [1, 4–6]. Initially, Gram-positive bacteria were thought not to produce EV because they lack outer membranes. However, Gram-positive bacterial EV were microscopically observed in 1990 [7], and now EV have been characterized from several infectious Gram-positive bacteria, including *S. aureus* [8], *B. anthracis* [9], *Listeria monocytogenes* [10], *Bacillus subtilis* [11], and *Clostridium perfringens* [12].

Gram-negative bacterial EV are already under development as vaccines and antibiotics, however the characterization of Gram-positive bacterial EV is still in the early stage. Further investigations of components and biological activities of Gram-positive bacterial EV are needed to better understand bacterial physiology and to develop therapeutic targets and applications. One characteristic that distinguishes several Gram-positive species is the propensity to form spores [13, 14] when nutrient levels fall. In the context of looking for mechanisms to control bacterial growth, it is of interest to ask if EV are formed during sporulation and what kind of cargo they carry.

High resolution microscopy and proteomics strategies are the major techniques used to characterize EV. Electron microscopy has been utilized to confirm the presence and purity of EV and also to visualize the shedding of EV from cells. MS-based proteomics and bioinformatics allow qualitative and quantitative characterization of the proteins of EV, which may in turn suggest biological activity and function [2]. In addition, fluorescence probes have recently emerged to analyze diverse interactions of EV with cells and organelles [2, 15]. We have used these techniques to ask whether *B. subtilis* shed EV during sporulation, if and how the EV protein content differs based on cellular stages, and how EV may interact with other *B. subtilis* cells. We have qualitatively and quantitatively examined the protein cargos of EV from vegetative and sporulating *B. subtilis* cells, seeking correlations with the distinctive process of sporulation.

## Methods

### Cell culture

To obtain vegetative cells, *B. subtilis* 168 cells (ATCC #23857) were grown in brain heart infusion (BHI) (Becton–Dickinson, Franklin Lakes, NJ). A sub-culture from a colony on BHI agar was inoculated into 500 mL BHI and incubated for 12 h (720 min) at 37 °C. Phase contrast microscopy indicated that sporulation had begun at 17 h, but not at 12 h. Cells were pelleted and supernatants that contained the EVs were collected at 12 h, as found previously to be optimal [11]. Three biological replicates were collected.

To induce sporulation, vegetative cell pellets were washed with PBS and resuspended in BHI-based sporulation medium (6 g BHI, 12 mg MnCl_2_, 4.8 g MgSO_4_, and 0.2 g CaCl_2_ in 500 mL) [16]. A culture time of 12 h was selected to obtain an optimal amount of EV. Cells in this medium were pelleted using the same conditions as for vegetative cells. Schaeffer-Fulton staining phase contrast microscopy [17] confirmed endospore formation in about 70 % of cells. The amounts of vegetative and sporulating cells were quantified as CFUs. Three biological replicates were prepared.

### Isolation and characterization of EV

EV were isolated from the supernatants of vegetative and sporulating cultures after 12 h, following methods published for Gram-positive bacterial EV with slight modifications [8, 9, 18]. Briefly, the supernatants were filtered through a 0.22 μm bottle-top vacuum filter (Corning) to remove remaining cell debris. EV were pelleted by ultracentrifugation at 150,000*g* for 90 min at 4 °C and washed with PBS (Beckman Coulter OptimaLE-80K ultracentrifuge with 70Yi rotor). Each pellet was resuspended in PBS. Finally the EV were washed three times with PBS on a 100 kDa Amicon filter. The resulting EV were stored at −80 °C until further use. Protein concentrations in purified EV were determined with a BCA protein assay kit (Pierce) according to the supplier’s instructions. To estimate lipid content, EV were stained with 5 µM DiO lipophilic dye (Invitrogen, Carlsbad, CA) in PBS for 1 h. Relative amounts of lipids were determined as a function of CFUs, using a F-4500 fluorescence spectrophotometer (Hitachi, Tokyo, Japan) at 498 nm excitation and 510 nm emission. Total intensities of solutions and unstained EV were used for background subtraction [19]. The EV were then characterized by electron microscopy. The EV population from the culture containing vegetative cells is referred to herein as vegetative EV, while that from the culture containing mostly sporulating cells is called sporulating EV. Three biological replicates of EV were prepared from vegetative and three from sporulating cells. These were processed and analyzed separately, and peptide identifications were combined within each phase type during the bioinformatic analysis.

### Transmission electron microscopy

For negative staining TEM, EV solutions (tenfold diluted) were applied to a carbon-coated formvar grid, which was covered by bacitracin (1 %) to spread the EV. The samples on the grids were washed and stained by 2 % uranyl acetate for 30 s. Images were obtained at 80 keV on a Zeiss EM10CA TEM (LEO Electron Microscopy, Thornwood, NY). Diameters of EV were sorted using Image J [20].

### Lysis of EV

Aliquots of vegetative and sporulating EV were lysed by three cycles of sonication on ice for 40 s in 50 mM NH_4_HCO_3_, pH 7.8, containing 8 M urea and 1× protease inhibitor cocktail (Sigma-Aldrich). The urea was then diluted to about 8 mM with 50 mM NH_4_HCO_3_. Protein concentrations were determined with a BCA protein assay kit (Thermo Fisher Scientific, Rockford, IL). Each lysate was reduced in 20 mM DL-dithiothreitol for 30 min at 56 °C and alkylated with 40 mM iodoacetamide in the dark for 30 min at room temperature. The samples were digested using Sequencing Grade Trypsin Gold (Promega) for 16 h at 37 °C with an enzyme to protein ratio of 1:30.

### HPLC–MS/MS

Tryptic peptides were analyzed on a Shimadzu Prominence nano-HPLC (Shimadzu BioSciences, Columbia, MD) in-line with a LTQ Orbitrap XL (Thermo Fisher Scientific). Peptides prepared from 1 µg protein were loaded onto an Acclaim PepMap 300 C18 precolumn (Dionex, Sunnyvale, CA) with 95 % solvent A (97.5 % H_2_O, 2.5 % acetonitrile, and 0.1 % formic acid) and 5 % solvent B (97.5 % acetonitrile, 2.5 % H_2_O, and 0.1 % formic acid) for 10 min. Peptides were fractionated on a C18 analytical column (300 Å, 150 × 0.15 mm, Grace Davison Discovery Sciences, Columbia, MD) using a linear gradient from 5 to 40 % solvent B for 120 min, followed by 40–85 % for 25 min. The flow rate was 500 nL/min. Precursor ions were analyzed by the orbitrap with a resolution of 30,000 at m/z 400. Product ion scans were acquired from the LTQ in a data-dependent mode (nine most abundant precursor ions, normalized collisional energy of 35). Three µscans were averaged per spectrum. A dynamic exclusion of 1 repeat count was applied over 180 s. Three technical injections were analyzed from each of three biological replicates.

### Bioinformatics

The PepArML meta-search engine [21] was used to identify peptides and proteins, combining results from seven search engines. A reference database of *B. subtilis* 168 with 4243 reviewed sequences was obtained from the UniProtKnowledgeBase (UniProtKB, January 2016). Carbamidomethylation of cysteine was set as a fixed modification with methionine oxidation as a variable modification. One missed cleavage was allowed. Precursor ion tolerance was ±2 Da and that of fragment ions was ±0.6 Da. Peptides identified in the nine vegetative and sporulating EV technical replicates (three from each of three biological replicates for each phase type) were combined and filtered at 1 % peptide-spectrum match (PSM) FDR [21, 22]. Filtered peptide identifications from vegetative and sporulating EV were used to infer proteins using a generalized parsimony analysis, in which two or more unshared peptides were required for protein identification, in either of, or both, vegetative and sporulating EV. Protein FDR of 4.95 % was estimated using decoy peptides carried through the parsimony analysis. Spectral counting was carried out after peptide identification filtering and parsimony analysis. Differences in spectral counts between vegetative and sporulating EV were determined as ratios of spectral counts, R_sc_ [23, 24]. Differential *p*-values of spectral counts were calculated by the Fisher exact-test and the χ^2^-test and corrected for multiple-testing using the Benjamini–Hochberg procedure. Cellular component, functional and biological process properties of identified proteins were evaluated with respect to the Gene Ontology (GO) and the Kyoto Encyclopedia of Genes and Genomes (KEGG) using the Protein Information Resource (PIR, pir.georgetown.edu January 2016) “batch retrieval” tool.

Relative abundances of proteins were expressed as R_sc_, which is the log_2_ ratio of spectral counts calculated for each protein from vegetative EV versus sporulating EV. Proteins with R_sc_ exceeding +1 or −1 and Fisher FDR values <0.05 were considered to have significantly greater abundances in their respective sample (>twofold). R_sc_ values were determined for all the proteins identified from vegetative and sporulating EV.

### Alkaline phosphatase activity

Enzymatic activities of alkaline phosphatases in EV were determined by Lowry’s colorimetric assay [25].

### Extracellular vesicle fusion

Interaction of EVs with *B. subtilis* cells was monitored using a self-quenching lipophilic dye, octadecyl rhodamine B chloride (R18, Invitrogen) [26, 27]. Sporulating EVs (25 µg proteins) were stained with 30 µM ethanolic R18 probes in staining buffer [27] for 1 h. R18-labeled EVs were washed twice and resuspended in 200 mM NaCl in PBS. *B. subtilis* cells grown on BHI agar were washed in PBS and resuspended in the washing buffer. The fluorescence of R18-labeled EVs was monitored on a F-4500 fluorescence spectrophotometer at 560 nm excitation and 590 nm emission with gentle shaking at room temperature. After equilibration of the EV suspension for 5 min, an aliquot of the cells was injected (1/5 by volume). The reaction was stopped by Triton X-100 at a final concentration of 0.3 %. The fluorescence was converted to percent of maximal fluorescence de-quenching (FD) following the equation %*FD* = [(*F* − *F*_*i*_)/(*F*_*max*_ − *F*_*i*_)] × 100, where *F* is the fluorescence intensity at each second, *F*_*i*_ is initial fluorescence of EV before the addition of cells, and *F*_*max*_ is maximal intensity after adding the detergent.

## Results and discussion

### EV secretion from *B. subtilis* at vegetative and sporulating phases

**Fig. 1:**
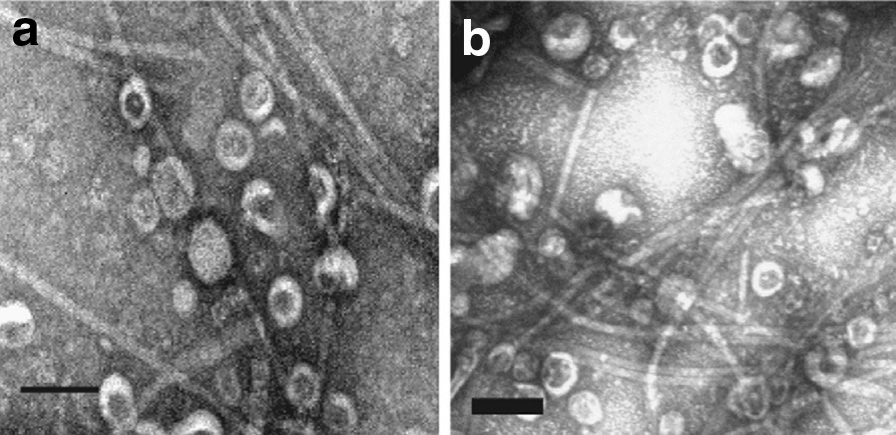
**a** Vegetative EVs, **b** sporulating EVs. The scale bars are 100nm

**Fig. 2:**
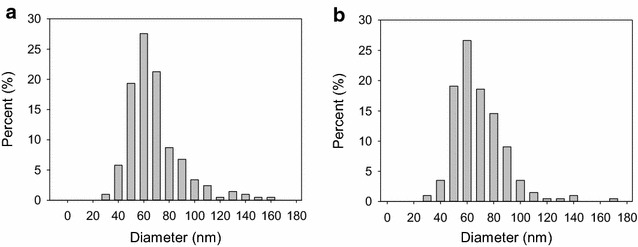
EV diameter estimations from TEM using Image J. **a** Vegetative EVs (n = 200), **b** sporulating EVs (n = 200)

The presence and purity of EV from vegetative and sporulating cell cultures were evaluated by negative staining and transmission electron microscopy (Fig. 1). We found that *B. subtilis* produces EV not only in the vegetative stage but also during sporulation. As in previous reports [11, 28] some flagella were isolated with both EV populations. Diameters of 200 EVs from each population were estimated using TEM images with Image J (Fig. 2). Both populations showed similar distributions centered around 60 nm. In order to compare the numbers of EV produced by comparable numbers of vegetative and sporulating cells, lipid content was determined as [6.7 ± 0.1] × 10^−7^ (DiO intensity/CFU, n = 3) for vegetative EV and [1.3 ± 0.4] × 10^−7^ (DiO intensity/CFU, n = 3) for sporulating EVs. Thus the size distributions of the two EV populations studied were similar to each other and within the range proposed for bacterial EVs [4, 29], while lipid content relative to CFU appeared to be slightly decreased in the sporulating condition.

### Characterization of EV proteins

**Fig. 3:**
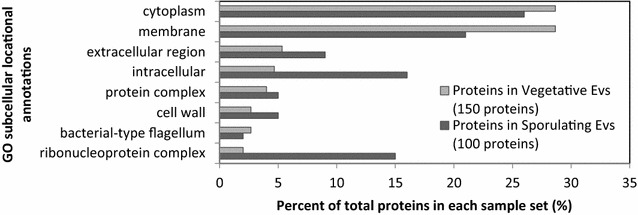
Subcellular locations of proteins in each EV sample referenced to parental cells. The most highly populated 7 locations from each are presented. Some proteins are assigned to more than one category

**Fig. 4:**
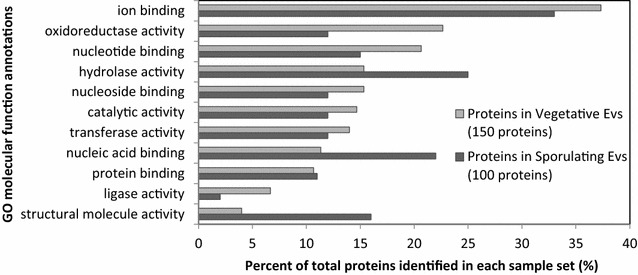
Biological processes of 150 proteins in vegetative EVs and 100 proteins in sporulating EVs. The ten most populated from each are shown. Some proteins are assigned to more than one category

One hundred and ninety-three proteins were identified in EV shed by vegetative and sporulating cells, after spectral FDR filtering at 1 % and parsimony analysis (Additional file 1: Table S1). Fifty-seven of these proteins were found in common in the two types of EV. Subcellular locations and molecular functions of the proteins in each sample were annotated based on GO annotations in the UniProtKB data base. Subcellular locations are compared in Fig. 3 where the largest numbers of proteins are assigned to membrane and cytoplasm in both groups. Sporulating EV contain a higher percent of proteins associated with the ribonucleoprotein complex. Distributions according to molecular function are shown in Fig. 4 with the largest number of proteins in both samples annotated for ion binding. Interesting differences include high contributions to oxidoreductase and nucleotide binding by proteins in vegetative EV, and to hydrolase activity, nucleic acid binding and structural activity in sporulating EV. Galperin et al. [30] have proposed that more than 12 % of all B.subtilis genes are expressed primarily during sporulation and point out that sporulation also affects other cellular processes significantly. Our observations indicate that such changes in protein synthesis are reflected in the contents of the EV as well.

### Proteins with significantly different abundances in vegetative and sporulating EV

Sixty-two proteins were found to be significantly more abundant (R_sc_ > 1 and Fisher-exact test FDR < 0.05) in vegetative EV than sporulating EVs, while 61 proteins were significantly more abundant (R_sc_ > 1 and Fisher-exact test FDR < 0.05) in sporulating EV (Additional file 2: Table S2). It was of interest to compare the proteins with significantly increased abundances in the two sets of EV. Functional annotations for more abundant proteins based on KEGG pathways were clustered using the PIR “batch retrieval” tool and are presented in Table 1. PIR provides five significant pathways for each group. As can be seen, proteins more abundant in vegetative EV are overwhelmingly associated with metabolism, while metabolic functions are reduced in proteins from sporulating EV and proteins associated with translation are prominent. This is consistent with Figs. 3 and 4 and may reflect a high level of translation associated with the early stages of sporulation [30]. It is relevant that abundant ribosomal and other translational proteins have been reported by others in spores themselves [31, 32].

**Table 1:** Enriched KEGG pathways annotated in the two EV samples using PIR

Enriched KEGG pathways of vegetative EV	Count	Fisher’s *p* value	Enriched KEGG pathways of sporulating EV	Count	Fisher’s *p* value
Metabolic pathways	26	0.02	Translation	13	0.01
Biosynthesis of secondary metabolites	17	0.02	Two-component systems	5	0.02
Microbial metabolism in diverse environments	14	0.02	Metabolic pathways	5	0.03
Glycolysis/gluconeogenesis	9	0.01	Microbial metabolism in diverse environments	4	0.01
Citrate cycle (TCA cycle)	6	0.01	ABC transporters	3	0.07

In the proteomic study reported here, about 30 % of the proteins identified in each EV type are shared in common. Although some vegetative cells remained in the sporulating culture when sporulating EV were harvested (see “Methods” section), the statistical validation of enhanced abundances of different proteins in the two EV populations reflects and confirms EV formation by sporulating cells. Many proteins associated with sporulation were identified in sporulating EV (Additional file 1: Table S1). Selected examples of proteins with statistically significant increased abundances are characterized in Table 2. Two of the enriched proteins—superoxide dismutase and stage O sporulation protein KE—are listed by Galverin and coworkers among the genes they consider essential for sporulation in *Bacillus* [30]. Spectral counting indicates that alkaline phosphatase III, a widely accepted marker of sporulation stage II [33], is enriched in sporulating EV, and chemical assays of alkaline phosphatase enzymatic activity (Additional file 3: Figure S1) support the spectral counting results. Septum site-determining protein DivIVA P71021 is also statistically enriched in sporulating EV. It has been shown to perform sporulation-specific functions required for asymmetric septation and activation of sporulation sigma factors [34]. Finally we note that at least one protein associated with antibiotic activity was demonstrated to be more abundant in sporulating EV. Polyketide synthase PKsM P40872 is part of the complex responsible for synthesis of bacillaene [35]. Secretion of this antibiotic has been proposed to complement sporulation as a survival strategy in wild type *B. subtilis* [36].

**Table 2:** Selected proteins with significant increase of abundance in sporulating EV (|Rsc| ≥ 1 and Fisher’s exact test FDR ≤ 5 %)

Uniprot Accession	Sporulation-associated proteins	R_sc_^a^	FDR	Biological process annotation
P19405	Alkaline phosphatase 3	7.1	2.32E−38	Metabolic process
P24137	Oligopeptide transport ATP-binding protein OppF (Stage 0 sporulation protein KE spo0KE)	5.8	9.84E−16	Sporulation
P71021	Septum site-determining protein DivIVA^c^	3.8	3.88E−10	Sporulation
P54375	Superoxide dismutase	2.2	2.63E−04	Stress response
P40872	Polyketide synthase PKsM	2.3	4.07E−02	Biosynthesis

^a^Spectral count ratio of sporulation EV proteins over vegetative EV proteins

### Fusion of EV with *B. subtilis* cells

In multiple studies of Gram-negative bacteria, proteins with antibiotic and other activities carried by EV have been shown to achieve intercellular effects by direct intercellular transfer (1,4,6). Similarly, proteins with antibiotic, enzymatic and pathologic activities carried in Gram-positive EV may be proposed to have intercellular effects, either through secretion or through direct intercellular transfer. Intercellular transfer of neither cargo nor activity was examined in the present work, however the feasibility of direct transfer is supported here by experiments that demonstrate fusion between EV and vegetative cells.

**Fig. 5:**
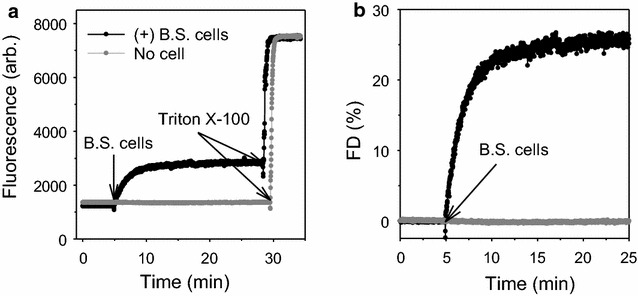
Fusion between EVs and vegetative *B. subtilis* cells. **a** Fluorescence detection of R18-labeled sporulating EVs in the absence of cells (*grey*) and with the addition of cells (*black*), followed by the disruption with Triton X-100. **b** Fluorescence de-quenching without (*grey*) and with cells present (*black*)

Fusion was demonstrated between *B. subtilis* cells and EV when sporulating EV were labeled with the self-quenching lipophilic probe R18. The R18 dyes are de-quenched upon dilution, as results from membrane fusion, and their fluorescence increases. The addition of vegetative cells to R18-labeled sporulating EV caused fluorescence to increase rapidly, indicating that membrane fusion occurred readily between EV and cells (Fig. 5).

## Conclusion

Extracellular vesicles have previously been purified from Gram-positive bacteria at the late exponential growth or stationary phases. In this study*, B. subtilis* was also observed to release EVs during sporulation, suggesting that EV biogenesis is a continuous process over the entire cellular life span. The production of EV by sporulating cells is further supported by the determination of significant differences in identities and abundances of the protein cargos of EV shed by stationary phase and sporulating *B. subtilis*. Strong differences were also documented in protein functions and KEGG pathways. The demonstration of the fusion of EV from Gram-positive cells in the present work lays the foundation for subsequent studies to confirm or not the transfer of protein activities to host cells or other bacteria in this manner.

## Additional files

10.1186/s12014-016-9107-z Proteins identified in sporulating (Spo) and vegetative (Veg) extra-cellular vesicles.
10.1186/s12014-016-9107-z Differentially abundant proteins in sporulating (Spo) and vegetative (Veg) extra-cellular vesicles.
10.1186/s12014-016-9107-z Alkaline phosphatase activities from EV proteomes (n=3).

## Abbreviations

BHI: brain heart infusion
DAVID: Database for Annotation, Visualization, and Integrated Discovery
FD: fluorescence dequenching
GO: Gene Ontology
KEGG: Kyoto Encyclopedia of Genes and Genomes
PIR: Protein Information Resource
pNPP: *p*-nitrophenyl phosphate
R18: octadecyl rhodamine B chloride
