# Supplementary material for: Extracellular vesicle proteomes reflect developmental phases of Bacillus subtilis
Source: Clin Proteomics. 2016 Mar 9;13:6. doi: 10.1186/s12014-016-9107-z (PMC4784445; doi:10.1186/s12014-016-9107-z)
Supplement: Supplementary file 3 — 10.1186/s12014-016-9107-z Alkaline phosphatase activities from EV proteomes (n=3). [file 12014_2016_9107_MOESM3_ESM.docx]

**Figure S1.** Alkaline phosphatase activities from EV proteomes (n=3).
